# Bacteriophages of *Gordonia* spp. Display a Spectrum of Diversity and Genetic Relationships

**DOI:** 10.1128/mBio.01069-17

**Published:** 2017-08-15

**Authors:** Welkin H. Pope, Travis N. Mavrich, Rebecca A. Garlena, Carlos A. Guerrero-Bustamante, Deborah Jacobs-Sera, Matthew T. Montgomery, Daniel A. Russell, Marcie H. Warner, Science Education Alliance-Phage Hunters Advancing Genomics and Evolutionary Science (SEA-PHAGES), Graham F. Hatfull

**Affiliations:** Department of Biological Sciences, University of Pittsburgh, Pittsburgh, Pennsylvania, USA; Harvard University

**Keywords:** *Gordonia*, bacteriophage genetics, bacteriophages

## Abstract

The global bacteriophage population is large, dynamic, old, and highly diverse genetically. Many phages are tailed and contain double-stranded DNA, but these remain poorly characterized genomically. A collection of over 1,000 phages infecting *Mycobacterium smegmatis* reveals the diversity of phages of a common bacterial host, but their relationships to phages of phylogenetically proximal hosts are not known. Comparative sequence analysis of 79 phages isolated on *Gordonia* shows these also to be diverse and that the phages can be grouped into 14 clusters of related genomes, with an additional 14 phages that are “singletons” with no closely related genomes. One group of six phages is closely related to Cluster A mycobacteriophages, but the other *Gordonia* phages are distant relatives and share only 10% of their genes with the mycobacteriophages. The *Gordonia* phage genomes vary in genome length (17.1 to 103.4 kb), percentage of GC content (47 to 68.8%), and genome architecture and contain a variety of features not seen in other phage genomes. Like the mycobacteriophages, the highly mosaic *Gordonia* phages demonstrate a spectrum of genetic relationships. We show this is a general property of bacteriophages and suggest that any barriers to genetic exchange are soft and readily violable.

## IMPORTANCE

Despite the numerical dominance of bacteriophages in the biosphere, there is a dearth of complete genomic sequences. Current genomic information reveals that phages are highly diverse genomically and have mosaic architectures formed by extensive horizontal genetic exchange. Comparative analysis of 79 phages of *Gordonia* shows them to not only be highly diverse, but to present a spectrum of relatedness. Most are distantly related to phages of the phylogenetically proximal host *Mycobacterium smegmatis*, although one group of *Gordonia* phages is more closely related to mycobacteriophages than to the other *Gordonia* phages. Phage genome sequence space remains largely unexplored, but further isolation and genomic comparison of phages targeted at related groups of hosts promise to reveal pathways of bacteriophage evolution.

## INTRODUCTION

Bacteriophages are the most abundant biological entities in the biosphere, with an estimated 10^31^ total particles (1). Investigations of viral sequence space and capsid structures suggest that the viral population is vast, dynamic, and old, containing large unexplored reservoirs of genetic information (2). The genetic texture of the phage population is ill defined, but the diversity is enormous, and the genomes are characteristically mosaic, with horizontal genetic transfer (HGT) mediated by illegitimate recombination playing an important role in their evolution (2, 3).

Tailed phages with double-stranded DNA (dsDNA) genomes predominate in the environment, and there are currently approximately 2,700 sequenced genomes in the GenBank nonredundant (nr) database. Phages of phylogenetically distal hosts typically share little or no DNA sequence similarity, and few if any gene products share amino acid sequence similarity (3). Patterns of diversity and evolutionary mechanisms can be explored by comparing phage genomes of closely related bacteria, and moderate-to-substantial (>20) collections of phage genome sequences have been determined for hosts such as mycobacteria (4), enterobacteria (5), *Staphylococcus* (6), *Pseudomonas* (7), *Bacillus*, *Arthrobacter* (61), and cyanobacteria (8). A large collection of phages known to infect a single common host, *Mycobacterium smegmatis*, shows high diversity, and they can be grouped in clusters based on overall nucleotide sequence similarity and shared gene content (9). Typically, two phages sharing nucleotide sequence similarity and gene content are placed in the same cluster, and phages in different clusters do not share extensive nucleotide similarity. Many of the clusters can be divided into subclusters based on comparisons of average nucleotide identity (ANI). Currently, there are over 1,360 sequenced mycobacteriophages grouped into 26 clusters (Clusters A to Z) and six singletons (those with no close relatives); 12 of the clusters are divided into subclusters. The number of phages in each cluster varies enormously (493 in Cluster A and 2 each in Clusters U, V, Y, and Z), and the large Cluster A is divided into 16 subclusters, illustrating the high diversity of this group of related phages. A detailed comparison of 627 of these genomes showed that phages in different clusters/singletons sometime share substantial numbers of genes—conferring a spectrum of diversity—as expected from the mosaic nature of the genomes. This continuum of diversity may be a common feature of phage genomes, although there are reported to be distinct lineages of cyanobacterial phages (8, 10) that are relatively isolated from genetic exchange with other phages. Mycobacteriophage subclusters, rather than clusters, and their counterparts in the enterobacteriophages (5) could be considered similar lineages, typically sharing over 90% of their genes.

The development of different genomic types is proposed to involve changes in host range that permit phages to migrate across the host landscape, such that different routes provide access to different parts of a large common gene pool (11). The mycobacteriophage Patience represents an example of a phage that appears to have entered the mycobacterial host neighborhood relatively recently, as it has a much lower percentage of GC content (50.3%) than *M. smegmatis* (67.3%); its overall codon usage profiles are also substantially different from those of its host (12). However, the codon usage profiles of highly expressed genes are more similar to those of *M. smegmatis* genes, suggesting that Patience is in the process of adapting to growth in this host (12). Deconstruction of the evolutionary histories of Patience and other mycobacteriophages is confounded by the lack of genomic characterization of phages of other bacterial hosts phylogenetically proximal to *M. smegmatis* (11).

To further explore these questions in phage diversity and evolution, we have comparatively analyzed 79 phages that infect *Gordonia* spp. (13–30). *Gordonia* and *Mycobacterium* are in the same phylum (*Actinobacteria*), order (*Actinomycetales*), and suborder (*Corynebacterineae*), and both contain mycolic acid-rich cell walls: *Gordonia terrae* 3612 and *M. smegmatis* mc^2^155 are closely related, with a 42% span-length match between complete genome sequences when using BLASTn. Members of *Gordonia* are aerobic heterotrophs, have been isolated from terrestrial and aquatic sources, including hydrocarbon-contaminated industrial sites, and have been implicated in opportunistic infections in immunocompromised individuals and foaming in wastewater treatment plants (31). The *Gordonia* phages are highly diverse and encompass a spectrum of genetic relatedness and mosaicism. However, only one subset of phages shows a close relationship to the mycobacteriophages, despite the phylogenetic proximity of their hosts and the similarities of the environments from which the phages were isolated (4, 32). This collection of *Gordonia* phages provides a resource for genetic analyses of *Gordonia* strains that evince considerable metabolic diversity (31) and include opportunistic pathogens (33).

## RESULTS

### Genometrics.

Sixty phages isolated on *Gordonia* spp. through enrichment culture or direct plating as part of the Science Education Alliance-Phage Hunters Advancing Genomics and Evolutionary Science (SEA-PHAGES) and the Phage Hunters Integrating Research and Education (PHIRE) programs (15, 18–28) were used for comparative analysis (Table 1), together with 19 *Gordonia* phages described previously (13, 16, 17). All but two of the SEA-PHAGES/PHIRE phages were isolated on *G. terrae* 3612; the other two were isolated on *Gordonia neofelifaecis* (34); the 19 previously described phages were isolated on *Gordonia* spp., including *G. terrae*, *G. rubripertincta*, *G. malaquae*, *G. sputi*, or *G. alkanivorans* (Table 1). These 79 phages span a wide range of genome lengths (17,118 to 103,424 bp) and percentages of GC content (47 to 68.8%), with many percentage of GC values distinct from those of their *Gordonia* hosts (*G. terrae* 3612, 67.8%). Most of the genomes have either defined ends with short (10 to 12 bases) 3′ single-stranded extensions or are circularly permuted and terminally redundant (Table 1). However, several have direct terminal repeats at their ends, varying in length from 191 bp to 1,182 bp (Table 1), an organization not seen in any of the sequenced mycobacteriophages. All of the 60 SEA-PHAGES and PHIRE phages were examined by electron microscopy, and all have icosahedral heads with flexible noncontractile tails and thus are morphologically grouped in the *Siphoviridae*; all of the 19 previously described *Gordonia* phages are also reported to be siphoviruses.

**TABLE 1:** *Gordonia* phage profiles

Phage name	Host species	Strain	Cluster	Length (bp)	GC %	Genome end^a^	Accession no.	Reference
JSwag	*G. terrae*	3612	A15	52,726	61.9	3′ 10-base ext.	KX557280	28
KatherineG	*G. terrae*	3612	A15	52,689	61.9	3′ 10-base ext.	KU998251	28
Remus	*G. terrae*	3612	A15	52,738	62.0	3′ 10-base ext.	KX557283	28
Rosalind	*G. terrae*	3612	A15	52,684	61.9	3′ 10-base ext.	KU998250	28
Soups	*G. terrae*	3612	A15	52,924	61.9	3′ 10-base ext.	KU998249	28
Strosahl	*G. terrae*	3612	A15	52,738	62.0	3′ 10-base ext.	KX557284	28
Bachita	*G. terrae*	3612	CQ1	93,843	61.9	3′ 10-base ext.	KU998247	28
ClubL	*G. terrae*	3612	CQ1	92,618	61.9	3′ 10-base ext.	KU998246	28
Cucurbita	*G. terrae*	3612	CQ1	93,686	62.0	3′ 10-base ext.	KU557276	28
Smoothie	*G. terrae*	3612	CQ1	93,139	61.9	3′ 10-base ext.	KU998244	28
OneUp	*G. terrae*	3612	CQ2	93,577	61.5	3′ 10-base ext.	KU998245	28
GRU1	*G. rubripertincta*	Grub38	CR1	65,766	65.5	Cir. Perm	JF923797	17
GTE5	*G. terrae*	Gter34	CR1	65,839	65.1	Cir. Perm	JF923796	17
GTE8	*G. terrae*	Gter34	CR2	67,617	66.0	?	KR053201	13
GMA7	*G. malaquae*	BEN700	CS1	73,419	56.6	?	KR063278	13
GTE7	*G. terrae*	Ben601	CS1	74,431	56.8	?	JN035618	30
Hotorobo	*G. terrae*	3612	CS2	76,972	58.9	191-bp DTR	KU963245	26
Monty	*G. terrae*	3612	CS2	75,680	58.9	191-bp DTR	KU998241	26
Woes	*G. terrae*	3612	CS3	73,752	59.1	184-bp DTR	KU998240	26
Benczkowski14	*G. terrae*	3612	CS4	75,380	59.5	1,172-bp DTR	KU963262	22
Demosthenes	*G. terrae*	3612	CS4	74,073	59.3	1,182-bp DTR	KU998242	28
Katyusha	*G. terrae*	3612	CS4	75,380	59.5	1,172-bp DTR	KU963258	22
Kvothe	*G. terrae*	3612	CS4	75,462	59.5	1,172-bp DTR	KU998243	28
Cozz	*G. terrae*	3612	CT	46,600	60.0	3′ 10-base ext.	KU998239	28
Emalyn	*G. terrae*	3612	CT	43,982	61.2	3′ 10-base ext.	KU963260	27
GTE2	*G. terrae*	Gter34	CT	45,540	60.3	?	HQ403646	16
Splinter	*G. terrae*	3612	CU1	45,858	66.1	3′ 12-base ext.	KU998238	28
Vendetta	*G. terrae*	3612	CU1	45,858	66.1	3′ 12-base ext.	KU998237	28
Gsput1	*G. sputi*	G11	CU2	43,505	62.8	5′ 16-base ext.	KP790011	14
Blueberry	*G. terrae*	3612	CV	54,990	67.0	3′ 10-base ext.	KU998236	28
CaptainKirk2	*G. terrae*	3612	CV	47,898	67.4	3′ 10-base ext.	KX557274	28
CarolAnn	*G. terrae*	3612	CV	54,167	66.9	3′ 10-base ext.	KX557275	28
Guacamole	*G. terrae*	3612	CV	49,894	67.2	3′ 10-base ext.	KU963259	18
Obliviate	*G. terrae*	3612	CV	49,286	67.5	3′ 10-base ext.	KU963254	18
UmaThurman	*G. terrae*	3612	CV	50,127	67.0	3′ 10-base ext.	KU963251	18
Utz	*G. terrae*	3612	CV	49,768	67.7	3′ 10-base ext.	KU998248	28
Jeanie	*G. neofelifaecis*	NRRL 59395	Cw1	17,118	68.6	3′ 10-base ext.	KU998256	28
McGonagall	*G. neofelifaecis*	NRRL 59395	Cw1	17,119	68.6	3′ 10-base ext.	KU998255	28
GMA5	*G. malaquae*	BEN700	Cw2	17,562	66.4	?	KR053198	13
GRU3	*G. rubripertincta*	Grub38	Cw2	17,727	66.5	?	KR053197	13
Kampe	*G. terrae*	3612	CX	80,649	47.0	249-bp DTR	KU998254	28
Orchid	*G. terrae*	3612	CX	80,650	47.0	249-bp DTR	KU998253	28
PatrickStar	*G. terrae*	3612	CX	80,729	47.0	249-bp DTR	KU998252	28
BatStarr	*G. terrae*	3612	CZ1	53,432	66.6	3′ 10-base ext.	KX557273	28
Kita	*G. terrae*	3612	CZ1	50,346	66.7	3′ 10-base ext.	KU963257	20
Nymphadora	*G. terrae*	3612	CZ1	53,431	66.6	3′ 10-base ext.	KU963255	20
Zirinka	*G. terrae*	3612	CZ1	52,077	66.7	3′ 11-base ext.	KX557287	28
Attis	*G. terrae*	3612	CZ2	47,881	66.8	3′ 11-base ext.	KU963247	24
SoilAssassin	*G. terrae*	3612	CZ2	47,880	66.8	3′ 11-base ext.	KU963246	24
BaxterFox	*G. terrae*	3612	CZ3	53,717	66.5	3′ 10-base ext.	KU963263	20
Yeezy	*G. terrae*	3612	CZ3	51,884	66.7	3′ 10-base ext.	KU963249	20
Howe	*G. terrae*	3612	CZ4	53,182	65.6	3′ 11-base ext.	KU252585	28
Bowser	*G. terrae*	3612	DB	46,570	67.1	3′ 10-base ext.	KU998235	15
Schwabeltier	*G. terrae*	3612	DB	46,895	67.0	3′ 10-base ext.	KU963252	15
Hedwig	*G. terrae*	3612	DB	44,536	67.2	3′ 10-base ext.	KX557279	28
Twister6	*G. terrae*	3612	DC	57,804	67.7	Cir. Perm	KX557286	28
Wizard	*G. terrae*	3612	DC	58,308	67.9	Cir. Perm	KU998234	28
GTE6	*G. terrae*	Gter34	DE	56,982	67.8	?	KR053200	13
Phinally	*G. terrae*	3612	DE	59,265	68.4	Cir. Perm	KU963253	19
Vivi2	*G. terrae*	3612	DE	59,337	67.1	Cir. Perm	KU963250	19
Gmala1	*Gordonia* sp.	G7	DF1	75,167	50.8	Cir. Perm	KP790009	14
GordDuk1	*Gordonia* sp.	G7	DF1	76,276	50.7	Cir. Perm	KP790010	14
GordTnk2	*Gordonia* sp.	G7	DF1	75,987	50.7	Cir. Perm	KP790008	14
GMA3	*G. malaquae*	BEN700	DF2	77,779	51.3	Cir. Perm	KR063279	13
Jumbo	*G. terrae*	3612	DF3	78,302	54.5	370-bp DTR	KX557281	28
Terapin	*G. terrae*	3612	Singleton	66,611	59.6	Cir. Perm	KX557285	28
Bantam	*G. terrae*	3612	Singleton	92,580	64.7	3′ 10-base ext.	KX557272	28
BetterKatz	*G. terrae*	3612	Singleton	50,636	67.1	3′ 10-base ext.	KU963261	23
BritBrat	*G. terrae*	3612	Singleton	55,524	65.0	3′ 10-base ext.	KU998233	28
Eyre	*G. terrae*	3612	Singleton	44,929	67.5	3′ 11-base ext.	KX557277	28
Ghobes	*G. terrae*	3612	Singleton	45,285	65.2	3′ 11-base ext.	KX557278	28
GMA1	*G. malaquae*	A448	Singleton	41,207	65.7	?	KR053195	13, 29
GMA2	*G. malaquae*	A448	Singleton	103,424	53.4	?	KR063281	13
GMA4	*G. malaquae*	BEN700	Singleton	45,537	66.4	?	KR053199	13
GMA6	*G. malaquae*	BEN700	Singleton	83,324	58.2	?	KR063280	13
GAL1	*G. alkanivorans*	DSMZ44369	Singleton	49,979	63.5	?	KR053194	13, 29
Lucky10	*G. terrae*	3612	Singleton	42,979	65.4	3′ 10-base ext.	KU963256	25
Nyceirae	*G. terrae*	3612	Singleton	41,857	67.5	3′ 9-base ext.	KX557282	28
Yvonnetastic	*G. terrae*	3612	Singleton	98,136	59.7	3′ 10-base ext.	KU963248	21

a ext., extension (single stranded); Cir. Perm, circularly permuted; DTR, direct terminal repeat.

All 79 *Gordonia* phages together with all sequenced phages known to infect hosts in the phylum *Actinobacteria* and deposited in GenBank as of October 2016 were used to construct a database (Actinobacteriophage_789) in the program Phamerator (35). Phamerator uses the alignment-free clustering algorithm kclust (4) to group genes into “phamilies” (phams) with related amino acid sequences. The 77,955 genes of the 789 phages form 11,035 phams with an average of 7 genes per pham; 4,630 of the phams are “orphams”: that is, they contain only a single gene.

### Grouping of *Gordonia* phages into clusters and subclusters.

**FIG 1:**
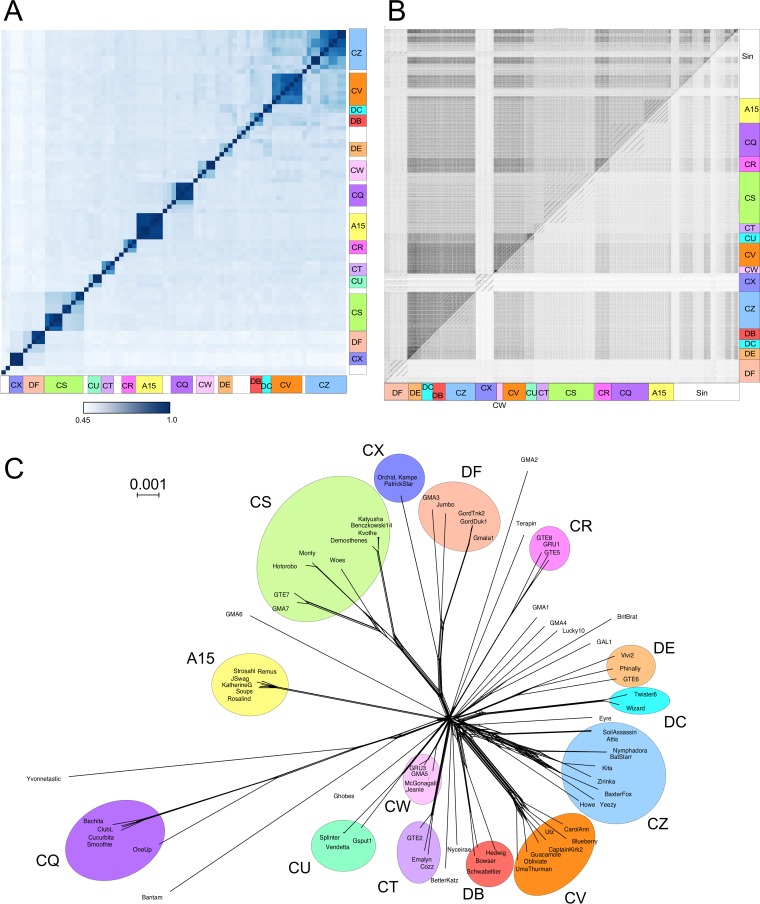
Analyses of complete genome sequences of phages of *Gordonia* spp. (A) Heat map of average nucleotide identities (ANIs) of *Gordonia* phages. Pairwise ANIs were calculated using DNA Master, and the heat map was generated using R. (B) Dot plot of complete *Gordonia* phage sequences. (C) SplitsTree network of shared gene content between *Gordonia* phages. All genes were grouped into phams using Phamerator. Each phage was scored by the presence/absence of phams, and the distance between each phage was calculated using SplitsTree. The scale bar indicates 0.001 substitution. Phages are colored according to cluster membership.

The *Gordonia* phages span considerable genetic diversity, and comparisons with average nucleotide identities (ANIs) and dot plots reveal small groups of phages that are closely related to each other at the nucleotide level, but which are not closely related to other groups (Fig. 1A and B). We initially assigned the *Gordonia* phages to clusters and subclusters using the same parameters as for the mycobacteriophages, forming 17 clusters and 19 singletons. However, this generates several instances in which phages of different clusters share large proportions of their genes and are likely to have common biological features. This is illustrated by the abundance of variable branch lengths in a SplitsTree network-based gene content phylogeny, creating examples where phages share a substantial portion of their gene content, without necessarily having nucleotide span-length matches exceeding 50% of the genomes (e.g., SoilAssassin and Yeezy [Fig. 1C]). We therefore chose to further refine the clustering parameters such that phages would be grouped into the same cluster if they share at least 35% of their genes (sorted into phamilies) with at least one other member of that cluster (Fig. 1 and Table 1). Thus, phages such as Attis and SoilAssassin that share 37 to 40% of their genes with Kita and BaxterFox are grouped together in Cluster CZ, whereas Orchid, Kampe, and PatrickStar (Cluster CX) share 28% of their genes with GMA3, Jumbo, GordTnk2, GordTuk1, and Gmala1 (Cluster DF), and clustered separately. These parameters place each of the phages in this analysis into one and only one cluster, however, it is conceivable that expansion of the collection with future isolates could yield a phage sufficiently similar to phages of two distinct clusters such as to warrant membership in both groups. Such an event would necessitate further refinement of our clustering parameters.

Using these revised parameters, 65 of the phages can be grouped into a total of 14 clusters, and the other 14 have no close relatives and are designated “singletons” (Table 1). Thirteen of the clusters contain only *Gordonia* phages, but a group of six *Gordonia* phages form a subcluster (A15) within the large group of Cluster A phages that previously exclusively contained mycobacteriophages (Table 1). Seven of the *Gordonia* phage clusters (CQ, CR, CS, CU, CW, CZ, and DF) can be subdivided into subclusters (Table 1). By comparison, when 60 mycobacteriophages had been sequenced, they were grouped into nine clusters (five with subcluster divisions) and five singletons (9), and when 80 were sequenced, they were grouped into 10 clusters (seven with subcluster divisions) and five singletons (36). Thus, at comparable sample sizes, the *Gordonia* phages are at least as diverse as the mycobacteriophages (28 clusters plus singletons for *Gordonia* phages versus 15 clusters plus singletons for mycobacteriophages). Genome maps of these phages are included in Fig. S1 to S27 at figshare (https://doi.org/10.6084/m9.figshare.5149663).

### A diverse array of systems for immunity and lysogenic maintenance in *Gordonia* phages.

The *Gordonia* phages form a variety of plaque types, ranging from clear to turbid, and the majority (53 of the 79) have genomic features associated with temperate phages, including tyrosine-integrase (Int-Y) or serine-integrase (Int-S), *parABS* partitioning systems, or repressors; only phages in Clusters CR, CS, CT, and DE and singletons Terapin, Ghobes, GMA2, and GMA6 lack these (Table 1). For 27 of the phages encoding a tyrosine-integrase (Int-Y), a putative *attP* common core sequence (20 to 40 bp) could be identified by similarity to a putative *attB* common core in the *G. terrae* 3612 genome (Table 2; see Table S1 at figshare). A total of 13 different *attB* sites were identified, 10 of which overlap host tRNA genes (Table 2). Interestingly, about half of these phages have the features of integration-dependent immunity systems described previously for mycobacteriophages (37), in which the *attP* site is located within the repressor gene (located immediately upstream), and the extreme C terminus of the repressor contains an *ssrA*-like degradation tag (Table S1). In some phages (e.g., Splinter and Vendetta) there are two potential *attP* sites corresponding to two different *attB* sites (Table 2; see Table S1 at figshare [https://doi.org/10.6084/m9.figshare.5149663]), but it is unclear if only one or both are used for integration. In GMA1, there is a single putative *attP* site, but it corresponds to two potential *attB* sites, suggesting that GMA1 can form double lysogens in which two prophages can be established in the same cell. We could not identify the *attP* site for about half of the Int-Y phages, but repressors of some phages (e.g., Nyceirae) have putative degradation tags and thus may also use integration-dependent immunity regulation. The *attP* sites for the serine-integrase (Int-S) phages could not be bioinformatically predicted as their *attP* and *attB* sites only have very short segments of sequence identity (38).

**TABLE 2:** Predicted *attB* sites used by *Gordonia* phages

*attB* site	tRNA	Locus tag^a^	Coordinates	Phage(s)
*attB-1*	tRNA^Ala^	BCM27_0045	11498–11475	GMA4
*attB-2*	tRNA^Arg^	BCM27_01925	422935–422970	UmaThurman, Twister6, Wizard
*attB-3*	tRNA^Ser^	BCM27_02280	499312–499338	Attis, SoilAssassin, Nymphadora, BatStarr, Kita
*attB-4*	tRNA^Thr^	BCM27_03985	854236–854279	CaptainKirk2, Guacamole, Obliviate, Hedwig, Eyre
*attB-5*	tRNA^Lys^	BCM27_06460	1424071–1424110	Splinter, Vendetta
*attB-6*	tRNA^Ala^	BCM27_07630	1682291–1682387	BaxterFox, Yeezy
*attB-7*	Intergenic	07635-07640	1683467–1683547	BaxterFox
*attB-8*	tRNA^Lys^	BCM27_10365	2294155–2294191	McGonagall, Jeanie, GMA5, GRU3
*attB-9*	tRNA^Arg^	BCM27_10435	2309527–2309565	Splinter, Vendetta, Bantam
*attB-10*	Intergenic	17750–17755	3953264–3953239	Howe, Lucky10
*attB-11*	tRNA^Ala^	BCM27_17805	3962309–3962284	Howe, Lucky10
*attB-12*	tRNA^Gly^	BCM27_22500	5080774–5080728	GMA1, GAL1
*attB-13*	Intergenic	22545–22550	5093356–5093312	GMA1, GAL1

a Locus tags are for *Gordonia terrae* 3612 (GenBank accession no. CP016594). Flanking locus tags are shown for intergenic sites.

**FIG 2:**
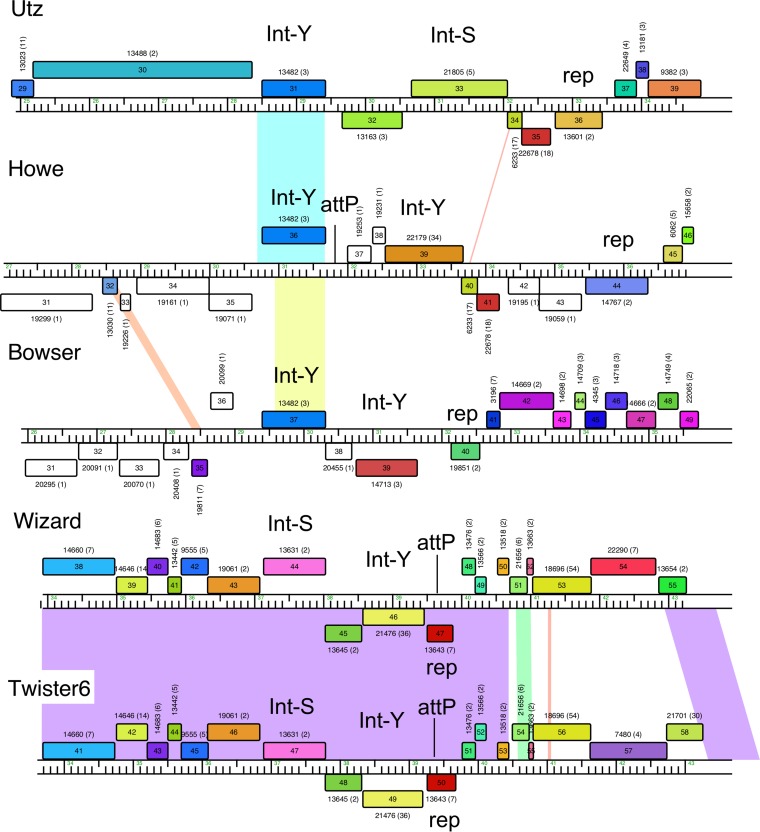
*Gordonia* phage genomes exhibit multiple integrases. Phamerator map of the integration cassettes of the six phages with double integrases. Integrases are labeled with catalytic residue (Y or S), and *attP* sites are indicated when known. Wizard gene *44* and Twister6 gene *47* are predicted to be nonfunctional.

Several phages are unusual in having two integrase-like genes located near the centers of their genomes, although some are partial genes (e.g., Twister6 gene *47* and Wizard gene *44*) and are likely to be defective (Fig. 2). For example, Wizard and Twister6 have seemingly intact integration-dependent immunity systems (37) but also have an Int-S-like gene that lacks the catalytic N-terminal domain (38) (Fig. 2). However, Utz, Howe, and Bowser all appear to have two intact integrase genes, all of which are Int-Y genes, except for Utz gene *33*; Utz gene *31*, Howe gene *36*, and Bowser gene *37* are closely related and share nucleotide sequence similarity (Fig. 2). Curiously, the *attP* site identified in Howe is absent from Bowser and Utz, such that even though the Int-Y proteins are related (71% amino acid identity), the integration system may be nonfunctional for lack of an *attP* site. Bowser’s second Int-Y has features of an integration-dependent immunity component and contains a putative *ssrA*-like tag at its C terminus, as does the repressor gene (gene *40*) (Fig. 2). However, we have not been able to identify a putative *attP* site either in the canonical locations within the repressor gene or elsewhere, and it is plausible that this integration system evolved to operate in a host other than *G. terrae*. We also note that the *attP* site associated with the Howe gene *36* Int-Y can potentially use two different *attB* sites (Fig. 2 and Table 2; see Table S1 at figshare). Finally, in Clusters CX and DF, the putative integrase genes are located in the middle of the right arm genes and lack the N-terminal domain associated with lambda-like Int-Y’s, and *attP* sites could not be identified. Although these systems are complex, it is not yet clear which of these recombinases mediate integration and which may be involved in recombination functions other than in phage integration (14). Taken together, the *Gordonia* phages show an unusual level of complexity of these site-specific recombinases that is not seen in phages of other actinobacterial hosts.

### Potential for prophage-mediated defense systems.

We have previously described a series of prophage-mediated defense systems encoded by Cluster N mycobacteriophages that defend against heterotypic viral attack (39). The genes conferring defense are located adjacent to the integration/immunity cassette in a region that is highly variable within a set of otherwise closely related genomes (39). Several groups of *Gordonia* phages share this genomic architecture, notably in Clusters CV, CZ, and DB (see Fig. S7, S10, and S11 at figshare [https://doi.org/10.6084/m9.figshare.5149663]). The phages within Cluster CV have 4 to 10 genes in the region between the minor tail protein genes and the integration/immunity systems, and although the functions of most of these are unknown, there are several with putative functions similar to those in Cluster N phages. These include Blueberry gene *33*, which possibly codes for a restriction protein, CaptainKirk2 genes *34* and *35*, and Utz genes *34* and *35* coding for toxin-antitoxin (TA) pairs, and genes (e.g., CaptainKirk2 genes *32* and *37*) coding for predicted membrane proteins. Likewise, in Cluster CZ phages, genes in this region code for candidate TA systems (e.g., Nymphadora genes *42* and *43* and Zirinka genes *33* and *34*) and membrane proteins (e.g., Kita gene *33*), and BaxterFox genes *31* and *32* are analogous to the MichelleMyBell viral defense genes *29* and *30*. Similarly, in the Cluster DB phages, there are 6 to 8 genes in this location, including genes for putative antitoxins (e.g., Bowser gene *35* and Schwabeltier gene *33*) and membrane proteins (e.g., Schwabeltier gene *35*); Bowser gene *31* codes for a putative lipase. Several other genomes also code for TA pairs, including Bantam and Eyre (see Table S2 at figshare). Taken together, we predict that the temperate *Gordonia* phages are replete with novel viral defense systems that warrant further investigation.

### Lysis systems in *Gordonia* phages.

Most mycobacteriophages code for three closely linked gene products involved in lysis—a holin, an endolysin (lysin A), and a mycolylarabinogalactan esterase (lysin B)—although some (e.g., Che12) lack the lysin B gene (40). Because the lysin B enzyme is responsible for cleavage of the mycobacterial outer membrane during lysis and because *Gordonia* cells contain mycolic acids, it is not surprising that most (all except for Clusters CR, CW, and DE and the singleton BetterKatz) of the *Gordonia* phages carry lysin B genes. However, there is great variation in where and how the lysis cassettes are organized. In many genomes (e.g., Cluster CR), the endolysin and holin genes are located downstream of the tail genes, a position where they are commonly located in the mycobacteriophages. However, in several phages (e.g., Clusters CV and CZ), they are found atypically within the minor tail protein genes. In a few genomes (e.g., Clusters CV, CZ, DB, DC, and DF), the lysin B genes are near the other lysis functions, but in most, they are displaced from it and are commonly positioned with the right arm genes. Finally, in some genomes (e.g., Cluster DF) the multiple domains found in mycobacteriophage endolysins (41) appear to be split into different genes.

### Atypically small *Gordonia* phage genomes.

**FIG 3:**
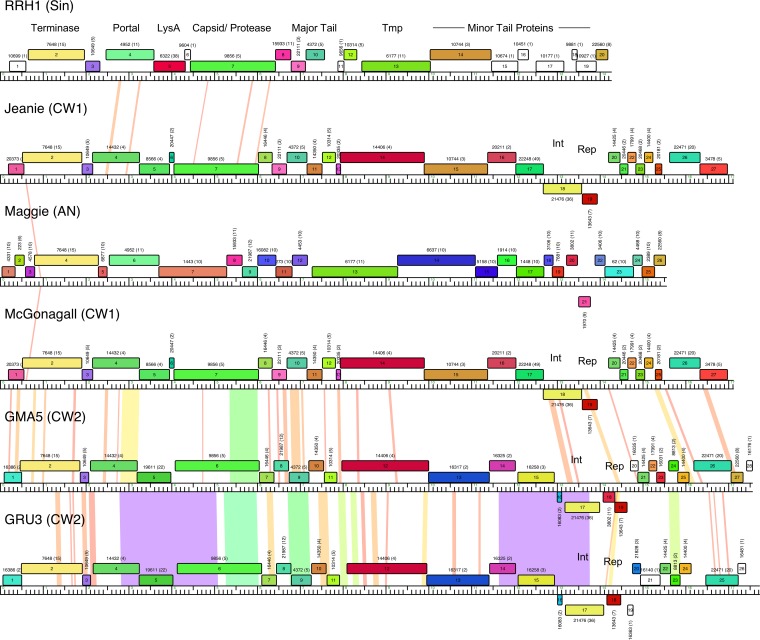
Small-genome actinobacteriophages. Shown are genomic maps of the small-genome phages included in the Actinobacteriophage_789 database. The central rule indicates nucleotide position—with large tick marks every 1 kbp—and the colored boxes indicate predicted genes. Genes are colored according to pham membership, with the number on the top of each box representing the pham number, followed by the number of members of the pham in the database in parentheses. Pairwise DNA sequence similarity calculated by BLASTn is shown between adjacent genomes and is spectrum colored, with violet being the most similar and red the least above a threshold E value of 10^−5^. Phages infect the following hosts: RRH1, *Rhodococcus* sp.; Jeanie, *G. neofelifaecis*; Maggie, *Arthrobacter*; McGonagall, *G. neofelifaecis*; GMA5, *G. malaquae*; and GRU1, *G. rubripertincta*.

The Cluster CW phages have genomes that are much smaller (17.1 to 17.7 kbp) than the smallest of the mycobacteriophage genomes (41 kbp), although phages with similarly sized genomes have been found for other actinobacterial hosts, including *Rhodococcus* (e.g., RRH1, 14.3 kbp) (42) and *Arthrobacter* (e.g., Maggie, 15.5 kbp) (61). All of these have siphoviral morphologies, and more than 80% of the genome coding capacity is devoted to the virion structure and assembly genes (Fig. 3). Although the small-genome phages of *Arthrobacter*, *Rhodococcus*, and *Gordonia* are not closely related, some of the gene products have amino acid sequence similarity, including all of the large terminase subunits (31 to 68% amino acid identity [Fig. 3]). Although RRH1 and Maggie are seemingly lytic, the *Gordonia* Cluster CW phages are temperate and form turbid plaques, and these carry integrase-dependent immunity systems as noted above. Although the repressors of GMA5 and GRU3 have diverged from those of Jeanie and McGonagall, all share an identical 37-bp *attP* site corresponding to an *attB* site overlapping a tRNA^Lys^ gene (BMC27_10365) in *G. terrae* 3612 (see Fig. S8 at figshare [https://doi.org/10.6084/m9.figshare.5149663]). GMA5 deviates somewhat from the canonical integration-dependent immunity system organizations in that an additional open reading frame (gene *18*) of no known function is inserted between the integrase and repressor genes (Fig. 3); a homologue is present in Maggie (gene *20*), although it lacks an immunity system (Fig. 3).

### Virion morphology variation in Cluster CZ.

**FIG 4:**
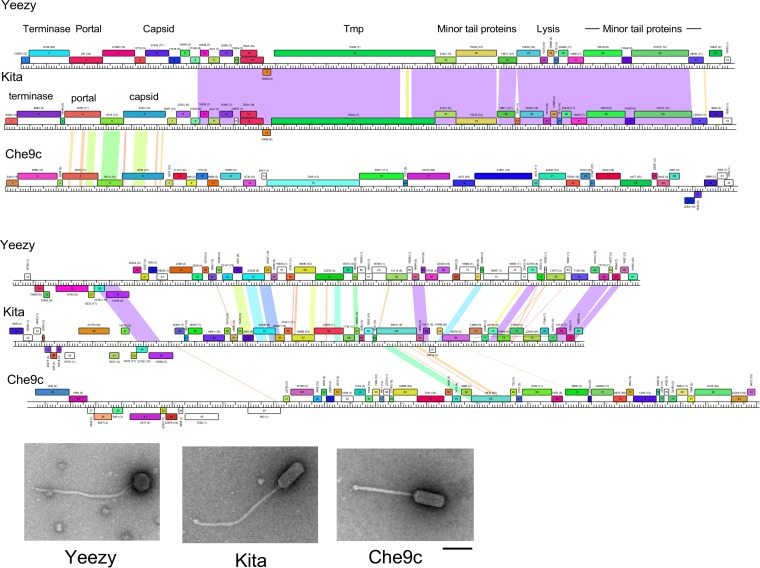
*Gordonia* phages in Clusters CZ exhibit multiple virion morphologies. (Top) Genome maps of *Gordonia* phages Kita and Yeezy and mycobacteriophage Che9c (see Fig. 3 for details) displayed in two tiers. Yeezy and Kita are both *Gordonia* phages but have different capsid morphologies: Yeezy is isometric, and Kita is prolate. Che9c is a mycobacteriophage that shares capsid structure and assembly genes with Kita and is also prolate. (Bottom) Electron micrographs of Kita, Yeezy, and Che9c. Scale bar = 100 nm.

*Gordonia* phage Cluster CZ is unusual in that it contains phages with distinctly different virion morphologies. All nine have long flexible noncontractile tails, but five (Howe, BaxterFox, Yeezy, Attis, and SoilAssassin) have isometric heads, whereas the other four (BatStarr, Kita, Nymphadora, and Zirinka) have prolate heads (Fig. 4). Genome comparisons show that the two sets of genomes differ in the leftmost eight genes that code for the terminase, portal, capsid maturation protease, and capsid proteins. Interestingly, the portal, protease, and capsid proteins of the prolate CZ phages have sequence similarity (65 to 67% amino acid identity) to homologues in the Cluster I mycobacteriophages, such as Che9c, which also have prolate capsids. This example illustrates a genetic link between the *Gordonia* and *Mycobacterium* phages and how the exchange of genes can result in morphological variation among phages with otherwise similar overall genomic architectures.

### Relationships of *Gordonia* phages to phages of other actinobacterial hosts.

**FIG 5:**
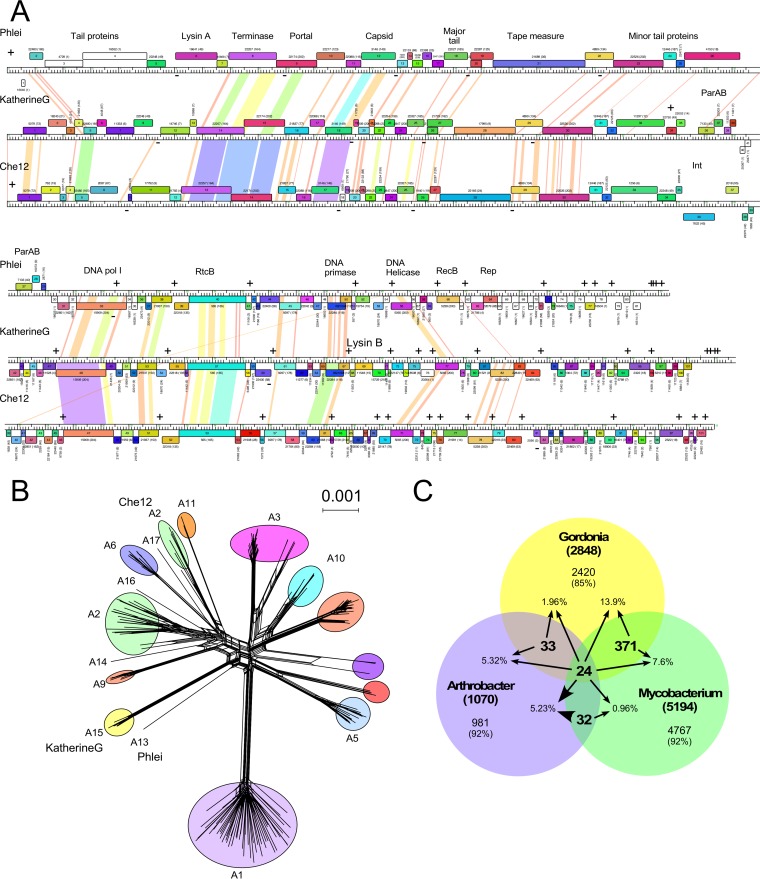
Relationships between *Gordonia* and *Mycobacterium* phages. (A) Genome maps of *Gordonia* phage KatherineG and mycobacteriophages Phlei and Che12 (see Fig. 3 for details). Stoperators are indicated with a + or − above or below the map to indicate sequence orientation. (B) SplitsTree (60) network representation of shared gene content among the Cluster A phages. Phage groups are colored according to subcluster. The positions of Che12 (A2), Phlei (A13), and KatherineG (A15) are shown. (C) Shared gene phamilies of phages of *Mycobacterium*, *Gordonia*, and *Arthrobacter*. Phamily membership was determined using Phamerator, and the proportions of shared phams were calculated: 24 phams out of 8,294 total are shared between phages of all three hosts, 395 phams are shared between *Gordonia* and *Mycobacterium*, 57 phams are shared between *Gordonia* and *Arthrobacter*, and 56 phams are shared between *Arthrobacter* and *Mycobacterium*. The percentages shown are determined relative to the total number of phams present in each host (the number in boldface in parentheses in each circle).

It is striking that six of the *Gordonia* phages (Table 1) fall into Cluster A—which previously contained solely mycobacteriophages—and form a distinct subcluster (A15) (Fig. 5; see Fig. S1 and Table S3 at figshare [https://doi.org/10.6084/m9.figshare.5149663]). The Subcluster A15 *Gordonia* phages are closely related to each other, and phage KatherineG shares the greatest sequence similarity with *Mycobacterium* phage Che12, Subcluster A2 (BLASTn score of 77% nucleotide identity spanning 47% of their genome lengths; they share 64% of their genes) (Fig. 5A and B). However, the *Gordonia* Subcluster A15 phages are distant relatives of all other *Gordonia* phages, and only about 15% of their genes are present in other *Gordonia* phages. The *Gordonia* Cluster A phage homologues are distributed broadly among the other *Gordonia* phages, including representatives of Clusters CQ, CS, CT, CU, CW, CZ, DB, DC, DE, and DF and singletons BritBrat, GMA4, GMA6, and Yvonnetastic.

The relationship of *Gordonia* Subcluster A15 phages to the mycobacteriophages is distinct and different from those of the other *Gordonia* phages (Fig. 5C). Of the 2,848 gene phamilies in the *Gordonia* phages, only 13.9% are shared with the *Mycobacterium* phages; the proportion of shared phamilies is only 10% if the A15 phages are excluded. Although few genes are shared, this is still greater than the number of phamilies shared with phages of the more distantly related host *Arthrobacter* (1.9% [Fig. 5C]), although this likely also reflects the differences in the total numbers of phages and phamilies that have been identified. As additional phages of different hosts within this phylogenetic space are characterized, further genetic connections between the phages are anticipated.

Cluster A is the largest group of related actinobacteriophages (192 in the database used here) and contains 16 subclusters (see phagesdb.org for a complete list of all current actinobacteriophage clusters and cluster membership). They are temperate (or recent lytic derivatives of temperate parents) and share overall genome architectures. They also have a common but unusual immunity regulation scheme in which the repressor binds to multiple binding sites (operators and “stoperators” [43]) located intergenically throughout the genomes. The Cluster A genomes all contain either an integration system using a tyrosine-integrase (44) or serine-integrase (45), although some from Subcluster A6 encode *parABS* partitioning systems (46, 47). The Subcluster A15 *Gordonia* phages are closely related to each other, all contain a *parABS* partitioning system, and the repressors are >99% identical to each other, indicating these likely form a homoimmune group. The A15 phages each contain 21 or 22 stoperator sites corresponding to the 13-bp asymmetric consensus sequence 5′-GGGGATTGTCAAG. The sequence conservation among the sites is similar to that reported previously (36, 43, 48), with positions 1, 9, 10, 11, 12, and 13 being invariant and positions 2 to 8 correlating with differences in immune specificity.

### *Gordonia* phages display a spectrum of relatedness.

**FIG 6:**
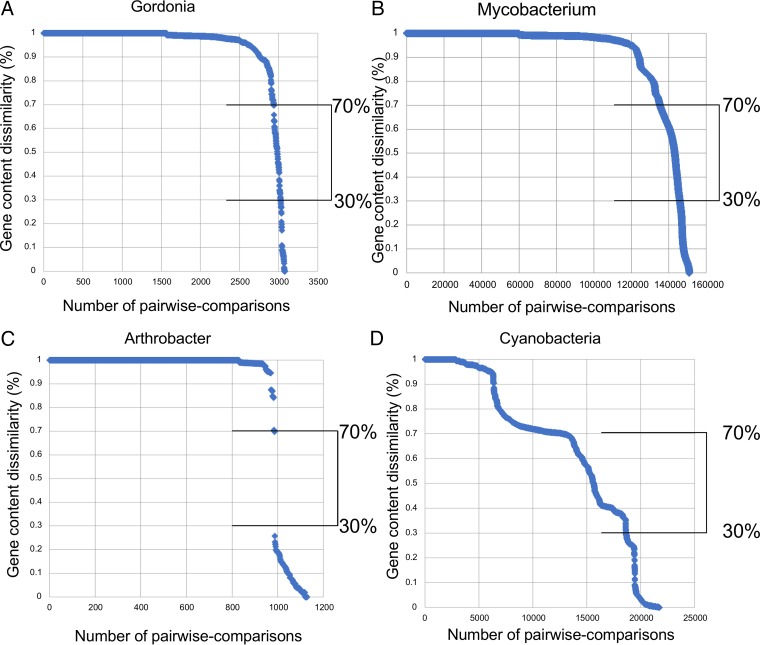
Gene content dissimilarity in phage populations. For all plots, the *x* axis is the ordered-by-magnitude individual pairwise comparisons, the *y* axis is the gene content dissimilarity (GCD [where 1 and 0 correspond to no shared genes and 100% shared genes, respectively]). (A) GCD in mycobacteriophages. (B) GCD in *Gordonia* phages. (C) GCD in *Arthrobacter* phages. (D) GCD in cyanophages. Both *Mycobacterium* and *Gordonia* phages exhibit a smooth curve of pairwise GCD values when ordered by magnitude. Cyanobacteriophages also exhibit a continuum of diversity with respect to GCD values; however, the slope of the line exhibits a number of plateaus, likely reflecting the “discrete” lineages described previously. *Arthrobacter* phages exhibit a large discontinuity, reflective of the few shared genes between clusters. Brackets indicate the interval between 30% and 70% gene content dissimilarity.

We noted above that the parameters used for cluster assignments were revised for grouping of the *Gordonia* phages, reflecting the lack of distinct boundaries between genome types and a spectrum of diversity as described for the mycobacteriophages (4). To explore the relationships between the *Gordonia* phages and other actinobacteriophages further, we calculated gene content dissimilarity (GCD) for pairwise genome comparisons between all 79 phages, where GCD is the proportion of shared phams relative to each genome’s total number of phams, averaging the two proportions and subtracting from 1 (see Materials and Methods) (49). Values range from 1, where no genes are shared (i.e., the two phages are 100% dissimilar) to 0, where the gene content is identical. The diversity of the *Gordonia* phages is illustrated by the range and distribution of GCD values (Fig. 6). A high proportion (88.6%) of pairwise comparisons have fewer than 10% shared genes (GCD, >0.9), with all 79 phages involved in at least one of the comparisons (Fig. 6). However, about 3% of the pairwise comparisons have values between 0.3 and 0.7 (i.e., reflecting 70% and 30% shared genes, respectively), and 38 of the 79 phages (47%) participate in at least one of these comparisons, representing 10 of the 28 groups (clusters plus singletons; 35.7%). Thus, although a value of 35% pairwise shared genes (GCD, 0.65) was used to group genomes into clusters, this is an arbitrarily chosen cutoff and not one that reflects a fundamental separation point among genome comparisons. It is helpful to note the cluster assignment parameters used here require 35% shared gene content with only one other phage, but because clusters (and subclusters) themselves can be quite diverse, the GCD values vary enormously within both clusters and subclusters.

Bacteriophages of *Arthrobacter* provide an informative comparison (Fig. 6). Pairwise comparison of 47 phages of *Arthrobacter* (61) shows greater delineation between groups that are closely related and those that are unrelated than for the *Gordonia* phages, (Fig. 6). For example, only 2 of the 1,129 (0.12%) pairwise genome comparisons have GCD values in the 30 to 70% range, contributed by just three (0.5%) of the phages, representing 2 of the 13 groups (16.7%) (11 clusters, two singletons). For the 550 mycobacteriophages in the data set used here, 7% of the pairwise genome comparisons have a GCD in the 30 to 70% range, and 80% of the mycobacteriophages—13 of the 30 groups (43%)—participate in at least one comparison; similar proportions were identified when we repeated the analysis using three random samplings of 80 mycobacteriophages. The parsimonious explanation is that the mycobacteriophages and *Gordonia* phages are exchanging genes at a higher rate with other phages of the same host than the *Arthrobacter* phages are. We similarly examined a group of cyanophages as these were reported elsewhere to form discrete lineages (8, 10). These have a more complex GCD pattern (Fig. 6), with 27% of the comparisons within the 30 to 70% bracket (Fig. 6), and 188 phages of 209 cyanophages participate in these comparisons.

### Phage genome relationships measured by MaxGCDGap.

**FIG 7:**
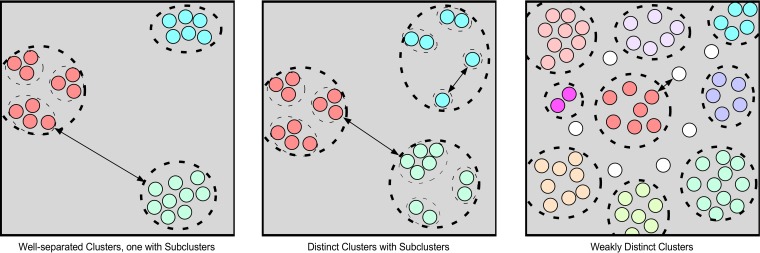
The various types of relationships of phage genomes are illustrated by three panels, varying (from left to right) from well-separated groups of related phages to a near continuum of relationships with weakly distinct groups. Phages sharing portions of their genomes can be grouped into clusters (shown in similar colors and surrounded by thick dashed circles), some of which can be subdivided into subclusters (surrounded by thin dashed circles). Typically, phages within a cluster have low pairwise gene content dissimilarity (GCD) values (i.e., they share a high proportion of their genes) and phages in different clusters have high pairwise GCD values. The relationships can be represented by MaxGCDGap values that correspond to discontinuities in the range of relationships of one phage relative to all others. Arrows indicate pairs of genomes in relationships at the high value of the MaxGCDGap parameter, which may occur between different clusters (and singletons) or between subclusters within a cluster. The MaxGCDGap values vary on the overall diversity within and between clusters, but are generally higher within phage populations with genetically well-separated phages (e.g., left panel) than where there is a near continuum of genetic diversity (e.g., right panel).

**FIG 8:**
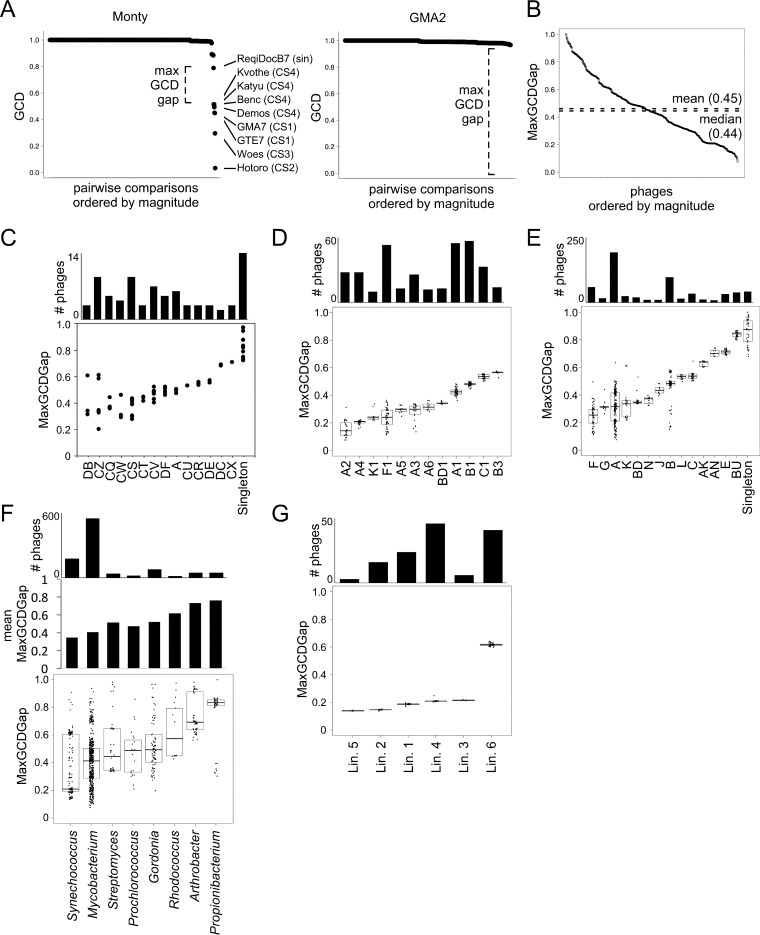
Phage genetic relationships as measured by MaxGCDGap. (A) Representative pairwise GCDs between *Gordonia* phage Monty (left) or GMA2 (right) and all other phages in the Actinobacteriophage_789 database, ordered by magnitude, similar to Fig. 6 (see Fig. S28 at figshare [https://doi.org/10.6084/m9.figshare.5149663]). Phages involved in comparisons with a GCD of <0.8 are highlighted. The maximum gap in GCD values (MaxGCDGap) was identified for each phage. For example, Cluster CS Monty’s MaxGCDGap occurs between *Gordonia* phage Kvothe (Cluster CS) and the singleton (sin) *Rhodococcus* phage ReqiDocB7. It should be noted that the second largest GCD gap is between phages in other CS subclusters (Hotorobo and Woes), illustrating that for some phages, the MaxGCDGap may reflect the distance between subclusters rather than clusters. Singleton phage GMA2 has no close relatives, and the MaxGCDGap is large and approaches 1.0. (B) All phage-specific MaxGCDGap values ordered by magnitude, with the mean and median indicated. Each data point represents a single phage genome. (C) All *Gordonia* phage-specific MaxGCDGaps grouped by cluster and ordered by median. Each data point represents a single phage genome. (D and E) Box plot distribution of actinobacteriophage-specific MaxGCDGaps from panel B grouped by subcluster (D) or cluster (E) and ordered by median. Boxes reflect the central 50% of the data, with the median as a black bar, and the individual MaxGCDGap values are superimposed. Only the most abundant groups are plotted. (F) Box plot distribution of phage-specific MaxGCDGaps as in panels D and E but grouped by host genus and with mean MaxGCDGaps displayed above. Only the most abundant genera are plotted. (G) Box plot distribution of *Synechococcus* phage-specific MaxGCDGaps as in panels D to F, grouped by the six previously identified lineages (Lin.) (8). The topmost bar chart in each panel indicates the number of phages per group.

To explore further the complex relationships between the *Gordonia* phages—and bacteriophage genomes in general—we developed an additional metric, the maximum gap in GCD values (MaxGCDGap). This is calculated by computing the pairwise GCD values for each phage against all other phages within a data set, ranking them by magnitude from largest to smallest, and calculating the difference between adjacent values (GCD gap) (see Materials and Methods) (Fig. 7 and Fig. 8A); the MaxGCDGap is the largest of these values. In contrast to other metrics of phage diversity used previously (4), the MaxGCDGap can be calculated for each phage within a data set without prior assignment to clusters, lineages, or other taxonomic groups and reflects a discontinuity in the relationships between one phage and all other phages (Fig. 7). It is important to note that the MaxGCDGap values are not absolute and depend on the size and composition of the data set analyzed. Moreover, the MaxGCDGap value is the difference between two GCD measures and is dependent on multiple parameters, including the variation within groups of related phages, the genetic relatedness of different groups of phages, and the extent to which the sampling reflects the larger populations. Nonetheless, groups of phages that are well separated in genetic sequence space are expected on average to have higher MaxGCDGap values than those in which there is little or only weak distinction between the groups (Fig. 7). Two examples are shown in Fig. 8A.

When all phages are rank ordered according to their MaxGCDGap values, an uninterrupted spectrum is observed around a MaxGCDGap mean of 0.45 (Fig. 8B), with values ranging from 0.076 to 1.0 (Fig. 8B). There is thus heterogeneity in the relationships, which are not constrained to any one proposed scenario in Fig. 7. When the *Gordonia* phages are examined and sorted by cluster (Fig. 8C) a broad range of values are observed, with the singletons having the highest values (most diverse from all other phages) and other phages ranging from MaxGCDGap values of 0.2 to 0.7 (Fig. 8C). A similar broad range of values is also observed with actinobacteriophages of other clusters and subclusters (Fig. 8D and E) and is reflected further when grouped by bacterial hosts (Fig. 8F). We note that phages of *Propionibacterium* (50) and *Arthrobacter* (61) have relatively high MaxGCDGap values and are examples of well-separated phage groups (Fig. 7), but in general, broad ranges of both mean values and distributions of values are observed for all other groups examined, reflecting the spectrum of values in the entire phage genome set (Fig. 8B). We note that phages of *Synechococcus* have both a relatively low mean MaxGCDGap value and a broad distribution, not substantially different from those of the mycobacteriophages. Overall, the observed patterns are consistent with a view in which the mosaic architecture of phage genomes results from HGT from a continuum of phage genetic space, albeit with substantially unequal sampling and undersampling of the individual phages from the population at large.

## DISCUSSION

Here we have described a collection of phages of *Gordonia* that expand our view of the overall diversity of the phage population and the relationships between phages of different hosts. The 79 phages encompass an amazingly large number of different genome types, but the relationships between them are complex. The genome architectures reveal many unexpected features, including atypical organizations of lysis genes, integration cassettes, and the possibility of many additional systems implicated in prophage-mediated viral defense.

We note that the grouping of *Gordonia* phages into clusters and subclusters is primarily a taxonomy of convenience (4, 5), recognizing the heterogeneity of the relationships and simplifying the discussion of phages that share many of their biological features. However, multiple lines of evidence (e.g., Fig. 1, 4, 6, and 8) show that many of the intracluster or intrasubcluster boundaries are ill-defined, because one or more phages within a cluster can share a substantial portion (up to ∼35%) of their genes with phages of other clusters. The cutoff values for grouping are thus largely arbitrary, but they are nevertheless intended to identify genomic relationships within a certain evolutionary scope to accommodate mosaicism. Thus, the groupings do not reflect definitive taxonomic or evolutionary boundaries between individual phages, and other cutoff values would expand or constrict group membership to highlight more distant or close genomic relationships, respectively. We predict strongly that isolation of additional *Gordonia* phages will further smooth their genetic landscape. This not only reflects the relationships illustrated by a large group of mycobacteriophages (4) but appears to be a general property of bacteriophage diversity and evolution (Fig. 8).

The complexities among the *Gordonia* phages warranted a revision of the clustering parameters used for mycobacteriophages (4, 9), and clustering of additional phages—especially of other host species—would benefit from a two-step process. First, genomes can be grouped using previously established parameters based on overall nucleotide similarity, which will suffice for some phage groups (e.g., *Arthrobacter* phages). However, if there the boundaries between genome types are less defined, the grouping can be further refined based on shared gene content, with any two phages sharing more than 35% of their genes being placed in the same cluster. We note that such parameters would impose few changes from the current clustering of mycobacteriophages, with the exception of Clusters I, P, and N (sharing 40 to 50% of their genes [4]), which would be grouped together. To avoid confusion, we do not propose a revision of the current mycobacteriophage clusters. We note again that the parameters for clustering are basically arbitrary and thus subject to further change with deeper sampling across the spectrum of diversity.

It is curious that one of the phage groups (Subcluster A15) is closely related to the mycobacteriophages, whereas the other *Gordonia* phages are distantly related to them and share few of their genes. This both indicates the value of characterizing phages of phylogenetically proximal hosts to identify genetic connections and the necessity to greatly expand the sizes of the phage collection on currently used hosts as well as additional hosts. The collection of >1,300 sequenced mycobacteriophages still reflects a considerable undersampling of the populations of the viruses at large, and we predict that it will be informative to continue isolating and sequencing *Gordonia* phage genomes until at least 1,000 have been characterized. Similar studies with phages of other hosts in the suborder *Corynebacterineae* targeting similar-sized collections will likely contribute greater numbers of genetic connections between the phages.

It was reported previously that *Synechococcus* phages form discrete lineages but participate in widespread HGT (8, 10). At least six lineages were defined by phylogenetic reconstruction of over 50 widely shared core genes common to the T4-like myoviruses (8). However, the MaxGCDGap values for five of these lineages are low (∼0.2 [Fig. 8G]), reflecting a low level of discontinuity across of the spectrum of possible genetic relationships and consistent with active participation in HGT (Fig. 7G). The degree of the discreteness of populations thus depends on the evolutionary time scale being considered. Over relatively short time frames, there clearly are distinct groups of phages (lineages of *Synechococcus* phages, equivalent to subclusters for the actinobacteriophages) that indicate either constraints on HGT or reflect the relative rates of HGT compared to the mutational clocks. However, for longer time frames, there is clearly extensive HGT among most of all phage genomes, albeit to various degrees depending on the hosts and the genome types.

The *Gordonia* phages present numerous opportunities for the development of genetic tools for *Gordonia* genetics. In particular, the broad range and types of integration systems will facilitate the development of integration-dependent vectors (44) for use in constructing recombinant *Gordonia* strains; the 13 predicted *attB* sites (Table 2) suggest the possibility of using multiple compatible vectors. The Subcluster A15 *parABS* partitioning systems may be used for stabilizing extrachromosomal plasmid vectors as they have been shown to do for the mycobacteria (46). There are also multiple examples of RecET-like systems in the *Gordonia* phages, and these have the potential to be used for *Gordonia* recombineering systems. Many of the phages are within a genome length and style that suggest they would be suitable for the construction of shuttle phasmids (51), which in turn could be exploited for the delivery of reporter genes and transposons (52). As noted previously (13), the lytic *Gordonia* phages or lytic derivatives of the temperate phages could be useful for controlling *Gordonia* growth in environment or biomedical applications.

Finally, many of the *Gordonia* phages are temperate and exhibit the genomic hallmarks associated with prophage-mediated viral defense systems (39). These could be explored by measuring plating efficiencies of *Gordonia* phages on different lysogenic strains, and we predict that the viral defense genes will be expressed in lysogeny. Given the genomic diversity, we anticipate that this will reveal many new systems that prophages use to defend against heterotypic phage attack.

## MATERIALS AND METHODS

Sixty phages were isolated on *Gordonia* spp. through enrichment culture or direct plating as part of the SEA-PHAGES and PHIRE programs, and dsDNA was extracted (15, 18–28). Double-stranded DNA genomes were sequenced using Illumina Mi-Seq and assembled using Newbler and Consed. The genomes were annotated using DNA Master (cobamide2.bio.pitt.edu), GeneMark (53), Glimmer (54), BLAST (55), Aragorn (56), tRNAscan-SE (57), and HHPred (58). All 79 *Gordonia* phages were analyzed with the program Phamerator (35) in conjunction with many other phages of the phylum *Actinobacteria* found in GenBank as of October 2016, for a total of 789 phage genomes (Phamerator database Actinobacteriophage_789). The 77,955 genes in the databases were grouped into 11,035 phamilies (phams) using Phamerator, which utilizes the alignment-free clustering algorithm kclust; this resulted in an average size of 7 genes per pham and 4,630 phams with single genes (orphams). A separate Phamerator database, Cyanobacteriophage_209, was constructed for phages infecting hosts of the phylum *Cyanobacteria*, involving a total of 209 published whole cyanophage genomes retrieved from the RefSeq and GenBank nr databases. Average nucleotide identity was calculated using DNA Master. The ANI heat map was generated by using “heatmap2” in R, and distance between genomes was calculated using the dist function with the “maximum” argument and the clustering method “single.” Phage order in the ANI figure is as follows: Nymphadora, BatStarr, Kita, Zirinka, BaxterFox, Yeezy, Attis, SoilAssassin, Howe, Eyre, Guacamole, CaptainKirk2, Obliviate, Utz, UmaThurman, Blueberry, CarolAnn, Wizard, Twister6, Bowser, Schwabeltier, Hedwig, Lucky10, BritBrat, GAL1, Nyceirae, Phinally, GTE6, Vivi2, BetterKatz, Jeanie, McGonagall, GRU3, GMA5, Ghobes, Smoothie, Bachita, ClubL, Cucurbita, OneUp, Yvonnetastic, Bantam, KatherineG, Soups, Rosalind, Strosahl, Remus, JSwag, GRU1, GTE5 GTE8, GMA4, GMA1, Emalyn, GTE2, Cozz, Splinter, Vendetta, Gsput1, Terapin, Hotorobo, Monty, Woes, GTE7, GMA5, Benczkowski14, Katyusha, Kvothe, Demosthenes, GordTnk2, Gmala1, GordDuk1, Jumbo, GMA3, Kampe, PatrickStar, Orchid, GMA2, GMA6.

Gene content networks were generated using SplitsTree (59, 60), based on pham membership of genes in each genome as calculated by Phamerator. Stoperators in the Subcluster A13 and A15 genomes were identified using the DNA Master scan function and searching for a 13-bp consensus sequence similar to the published A1 and A2 consensus sequences with similar instances and orientations as those sequences. For Che12 (A2), the consensus sequence is 5′-GGTGGTTGTCAAG, for Phlei (A13) the sequence is 5′-GCTTGGGTGTCAAG, and for KatherineG (A15), the sequence is 5′-GGGGATTGTCAAG. No more than 2 substitutions from the consensus sequence per sequence instance were allowed to be identified as a stoperator.

All pairwise gene content dissimiliarities (GCDs) were calculated for each phage database using custom written python scripts (49). GCD is calculated by determining the proportion of shared phams relative to each genome’s total number of phams, and then averaging the two proportions:

$$\text{GCD}=1-\left(\frac{\frac{\text{Shared phams}}{\text{Total phams in genome A}}+\frac{\text{Shared phams}}{\text{Total phams in genome B}}}{2}\right)$$

GCD plots were generated in R or Excel. Phage-specific MaxGCDGaps were calculated using custom written python scripts, as follows. For each phage, all pairwise GCD values were ranked by magnitude, and the difference between each consecutive GCD value was calculated (GCD gap). GCD gap is calculated as follows:

$${\text{GCD}}_{n}-{\text{GCD}}_{n\text{+1}}=\text{GCD }{\text{gap}}_{\left(n,n+1\right)}$$

The maximum GCD gap of the data subset was identified, which can range from near 0 (indicating small gene content discontinuities) to 1 (indicating large gene content discontinuities) (see Fig. S28 at figshare [https://doi.org/10.6084/m9.figshare.5149663]). Box plot distributions of MaxGCDGaps were plotted in R. All scripts are available upon request.

For electron microscopy, phage lysates were applied to carbon-coated copper grids, stained with 1% uranyl acetate, and imaged using a Morgani Technai transmission electron microscope.
